# Detection of Shiga Toxins by Lateral Flow Assay

**DOI:** 10.3390/toxins7041163

**Published:** 2015-04-03

**Authors:** Kathryn H. Ching, Xiaohua He, Larry H. Stanker, Alice V. Lin, Jeffery A. McGarvey, Robert Hnasko

**Affiliations:** 1 Produce Safety & Microbiology Research Unit, Agricultural Research Service, U.S. Department of Agriculture, 800 Buchanan St, Albany, CA 94710, USA; E-Mails: kathryn.ching@ars.usda.gov (K.H.C.); alice.lin@ars.usda.gov (A.V.L.); 2 Foodborne Toxin Detection and Prevention Research Unit, Agricultural Research Service, U.S. Department of Agriculture, 800 Buchanan St, Albany, CA 94710, USA; E-Mails: xiaohua.he@ars.usda.gov (X.H.); larry.stanker@ars.usda.gov (L.H.S.); jeffery.mcgarvey@ars.usda.gov (J.A.M.)

**Keywords:** shiga toxin, Stx, toxin, STEC, *E. coli* O157, immunoassay, lateral flow assay, food safety

## Abstract

Shiga toxin-producing *Escherichia coli* (STEC) produce shiga toxins (Stxs) that can cause human disease and death. The contamination of food products with STEC represents a food safety problem that necessitates rapid and effective detection strategies to mitigate risk. In this manuscript, we report the development of a colorimetric lateral flow assay (LFA) for the rapid detection of Stxs in <10 min using a pair of monoclonal antibodies that bind epitopes common to Stx1 and six Stx2 variants. This LFA provides a rapid and sensitive test for the detection of Stxs directly from STEC culture supernatants or at risk food samples with a 0.1 ng/mL limit of detection (LOD) for Stx2a. This Stx LFA is applicable for use in the rapid evaluation of Stx production from cultured *E. coli* strains or as a tool to augment current methods as part of food safety testing.

## 1. Introduction

*Escherichia coli* (*E. coli*) are part of the normal flora of the mammalian gastrointestinal tract and are largely non-pathogenic. The expression of certain virulence factors and genetic loci by strains of *E. coli* can dramatically alter this relationship, resulting in infection and disease in animals. Shiga toxin-producing *E. coli* (STEC) are a group of bacteria that produce shiga toxins (Stxs) responsible for illness in >200,000 individuals each year in the United States through foodborne contamination [1,2]. Cattle are the largest reservoir of STEC in North America, and outbreaks are frequently caused by consumption of under-cooked ground beef, milk products or through the spread of contaminated manure on leafy greens via the water supply [3,4,5,6]. Infection by STEC can cause hemorrhagic colitis (HC), characterized by fever and bloody diarrhea that can develop into hemolytic uremic syndrome (HUS), resulting in acute kidney failure and death [7]. *E. coli* O157:H7 has historically been the most commonly-reported serotype in outbreaks of HUS in the U.S. [8], and detection takes advantage of its sorbitol-negative phenotype, allowing identification using sorbitol-MacConkey plates (SMAC) as part of standard food safety testing [2,9,10]. Stx-producing non-O157 strains, which are increasingly associated with major disease outbreaks, cannot be detected by the SMAC method, as they lack the sorbitol-negative phenotype, and their emergence has necessitated the development of alternative means of detection [11].

Stxs are members of the AB_5_ toxin family. The holotoxin is composed of two major groups, Stx1 and Stx2, which share 56% sequence homology [12]. Stx1 exposure is generally associated with mild clinical symptoms, while variants of Stx2 are associated with more severe diseases [13]. Renal tubular and vascular epithelial cells are particularly sensitive to the Stx holotoxins, as they express high levels of cell surface globotriaosylceramide (Gb3) and globotetrasylceramide (Gb4) receptors, which bind to Stx B-subunits, resulting in Stx entry into the cytoplasm. Intracellular Stx inactivates ribosomes using the *N*-glycosidase activity of the A-subunit, resulting in blocked protein translation and cell death [14].

A very small number of STEC bacteria are sufficient to cause illness, and procedures that prevent contamination of food remain pivotal to any commercial producer, as the impact of an outbreak can be catastrophic [15]. Current methods used in industrial food testing rely on complex workflow strategies, sophisticated instrumentation and highly trained experts to assure food quality and safety. Despite intensive hygiene procedures used during food production, the complexity of the supply chain involving time-limited agricultural products can result in the introduction of STEC contamination at many points during processing. Proper “test and hold” methods used at the end of production are effective in identifying STEC-contaminated product and limiting distribution responsible for outbreaks. However, added quality assurance testing that could be used repeatedly throughout the supply chain to test for potential STEC contamination would limit downstream impact on production and product.

Lateral flow assays (LFAs) are simple to use diagnostic tools that are designed to be implemented in hazard analysis and critical control point (HACCP) systems of food manufacturers and producers. These rapid and cost-effective food safety tests can be deployed with minimal training along the supply chain to augment more robust food safety measures and maintain quality assurance from farm to plate. The LFA has shown steady growth in point-of-care settings for disease diagnosis, as tools for the identification of biothreat agents by first responders in the field and in industrial food production to monitor instrument hygiene and food contamination [16,17,18]. The generation of high-affinity monoclonal antibodies to target analytes combined with new reporter molecules with enhanced signal generation and portable hand-held detection capability has resulted in LFAs with quantitative capabilities and sensitivities equivalent to traditional plate-based sandwich enzyme-linked immunoassays (sELISA) [19].

In a previous report, we characterized several monoclonal antibodies generated against a non-toxic recombinant Stx2a toxoid and described a sensitive ELISA using a pair of mAbs for Stx2 detection [20]. In this report, we develop a colorimetric LFA that directly targets the etiological Stx agent for detection. The Stx LFA uses our monoclonal antibody Stx2-1 (IgG_1_/κ, K_D_ = 1.3 nM, A-subunit binding) for Stx capture and Stx2-2 (IgG_2A_/κ, K_D_ = 0.71 nM, A- and B-subunit binding) as a gold-conjugated detector mAb. Epitope mapping of these mAbs suggests that they bind discontinuous epitopes of the Stx holotoxin. There are four FDA-approved Stx immunoassays; two are available in the LFA format (Merck and Meridian Biosciences) with manufactures reporting sensitivities from 1.25 ng/mL to 62.5 ng/mL for Stx1 and Stx2. These tests have been validated on culture supernatants from STEC strains, but it is unclear if they can identify all Stx2 variants [21]. Here, we demonstrate the ability of our device to detect Stx1 and six variants of Stx2 from STEC culture supernatants and at-risk food matrices. This Stx LFA can provide rapid detection of Stxs directly from food-sampled bacterial cultures or “at-risk product” to improve food safety.

## 2. Materials and Methods

Antibodies: Stx2-1 and Stx2-2 mAbs were affinity purified using protein-G sepharose (Sigma, St. Louis, MO, USA) from ascites generated in Balb/cJ mice, and stock solutions were diluted to 2 mg/mL in PBS (pH 7.0). Stx2-1 was used to create the test (T) line, and Stx2-2 was actively conjugated to 40-nm gold particles using the InnovaCoat gold labeling kit according to the manufacturer’s instructions (Innova Biosciences, Cambridge, UK), resulting in a gold-labeled Stx2-2 mAb at OD_40_. Cross-adsorbed AffiniPure donkey anti-mouse IgG was obtained from Jackson ImmunoResearch (West Grove, PA, USA) and was used to generate the LFA control (C) line.

STEC cultures and Shiga toxins (Stx): Purified Stx1 and Stx2a were purchased from List Biological Laboratories (Campbell, CA, USA). Purified Stx2a was quantitated by BCA assay and used to determine assay sensitivity and to spike in different food matrices. The STEC strains used in these experiments have been previously characterized and are identified in Table 1 [22]. Culture supernatants were collected during the stationary phase by centrifugation (more toxins in the supernatant at this phase) and stored at −80 °C until use. Prior to application on the LFA, supernatants were diluted 1:5 in 10 mM PBS.

Construction of the lateral flow device: Purified Stx2-1 and a donkey anti-mouse IgG were each diluted to a concentration of 1 mg/mL in 10 mM PBS containing 3% methanol. The diluted antibodies were separately immobilized on Immunopore SP membrane (GE Healthcare, Pittsburgh, PA, USA) using a BioJet Quanti Liquid Dispensing System (BioDot, Irvine, CA, USA) at a rate of 1 µL/cm in 40-nL drops. The distance between the proximal end of the test strip and the test line was 12 mm. The control line was immobilized 4 mm distal to the test line. The membrane was dried at 37 °C, then blocked in 10 mM PBS containing 1% BSA (IgG-free, protease free, Jackson ImmunoResearch, West Grove, PA, USA) and 0.5% Triton X-100. The membrane was then washed twice with 5 mM Na_2_HPO_4_ containing 0.01% TX-100, followed by a final wash in dH_2_O. The membrane was then dried and affixed to a PVC backing card (Diagnostic Consulting Network, Carlsbad, CA, USA). The liquid absorbent sink, a cellulose/glass fiber pad (CF6, GE Healthcare, Pittsburgh, PA, USA), was affixed 5 mm from the control line and had an approximately 2-mm overlap with the membrane. Test strips were cut to a 5-mm width. To initiate the assay, 50 µL of analyte diluted in 10 mM PBS were added to a glass test tube containing 2 µL gold-labeled Stx2-2 (OD 40), mixed briefly, and a test strip was dropped directly into the tube. The assay test line (T) began to resolve within 2 min and was complete by 10 min.

**Table 1:** Characterization of shiga toxigenic *E. coli* (STEC) strains. Nd, not determined.

*Stx.* Genotype	Strain	Serotype	Origin	Reference	Cytotoxicity (CD_50_)
Stx1	RM7375	O26	Human	This Study	Nd
Stx2a	RM10638	0157:H7	Cow (2009)	[22]	0.10 ng
Stx2b	RM7005	O118:H2	Clinical	[23]	Nd
Stx2c	RM10058	O157:H7	Bird (2009)	[22]	1.00 ng
Stx2d	RM8013	Nd	Cow (2008)	[22]	1.00 ng
Stx2e	RM7110	O139:NM	Pig	[24]	Nd
Stx2f	RM7007	O128:H2	Feral pigeon	[25]	Nd
Stx2g	RM10468	Nd	Cow (2009)	[22]	0.01 ng
Stx-negative	RM4876	O157:H7	Watershed (2005)	[26]	Nd

Preparation of spiked food matrices: Lettuce, 90%-lean ground beef and milk (1%, 2% and non-fat) were obtained from a local grocer and stored at 4 °C until use. Samples of 0.1 g of lettuce or beef were mixed with 1 mL 10 mM PBS and spiked with purified Stx2a toxin in a microcentrifuge tube. Samples were vortexed extensively, then centrifuged at 12,000× *g* for 12 min at 4 °C. The resulting supernatant was then removed and tested directly. For milk samples, 1-mL aliquots were spiked with toxin, then either diluted 1:10 in the corresponding milk and tested directly or samples were centrifuged at 12,000× *g* for 12 min at 4 °C and the resulting supernatant tested.

Imaging and densitometry: Resolved LFA strips were photographed using a BioDot test (BioDot Inc., Irvine, CA, USA) strip reader (TSR3000) with a flat field camera. The resulting images were converted to 8-bit grayscale, saved in TIFF format and analyzed using ImageJ software (NIH, Bethesda, MD, USA). Densitometry was performed using a constant rectangular area that integrated background noise above and below the center at the test line for each strip from three independent experiments. Data analyses were performed using SigmaPlot (Systat Software, San Jose, CA, USA) and expressed as the average density of the test line ± SEM.

## 3. Results

**Figure 1:**
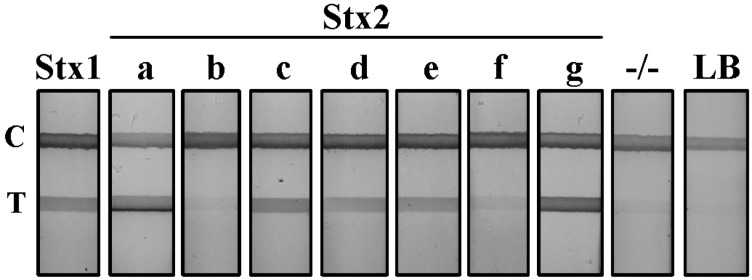
Detection of shiga toxins from STEC serotypes by lateral flow assay (LFA). Supernatant from Stx1 and seven variant Stx2-producing STEC cultures were diluted 1:5 in PBS and evaluated using the Stx LFA. −/−, a non-Stx-producing *E. coli* serotype; LB, Luria broth; T, test line; C, control line.

Stx LFA specificity: To determine the binding specificity of our Stx LFA, we evaluated nine independent STEC strains (Table 1). Genetic analysis confirmed that each strain carried only one Stx variant gene, and toxin expression was evaluated by the Vero cell assay [22]. Culture supernatants from each STEC strain along with non-STEC *E. coli* controls were diluted and positive LFA test lines resolved for those producing Stx1 and Stx2 variants (Figure 1). The supernatant from Stx1-, Stx2a- and Stx2g-producing cultures gave a strong positive result on the LFA with a dense test line resolving <1 min after initiation of the assay. Culture supernatants from Stx2c-, 2d-, 2e- and 2f-producing *E. coli* strains were slower to resolve by LFA and resulted in test lines of weaker intensity. The detection of the Stx2b variant in the supernatant of the O118:H2 clinical serotype was inconclusive. The Stx2b test line resolved at an intensity that was equivalent to, or slightly above, the background established by non-Stx producing *E. coli* culture supernatant (−/−). Luria-Bertani broth (LB) served as a negative test line control. All LFA test strips displayed a visible control line, validating the proper function of the assay. The presence of these toxins in the culture supernatant was confirmed by a sandwich ELISA using a polyclonal anti-Stx antibody (unpublished data).

**Figure 2:**
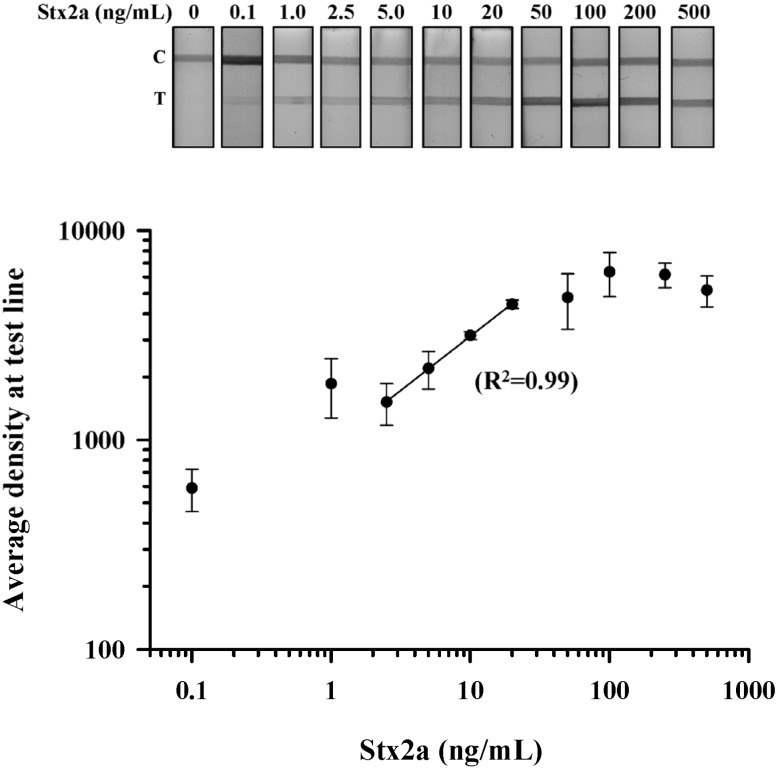
Dose-dependent detection of purified Stx2a by LFA. Purified Stx2a was serially diluted in PBS from 500 to 0.1 ng/mL and assessed by LFA (**top panel**); Density measurements were obtained at the test line from three independent experiments and the data plotted as the mean (±SEM) at the test line for each Stx2a dilution (**bottom panel**). Regression analysis revealed a linear relationship between the density of the test line and the concentration of Stx2a between 20 and 2.5 ng/mL (*R*^2^ = 0.99). T, test line; C, control line.

Stx LFA sensitivity: Purified Stx2a was used to establish the limit of detection (LOD) for the LFA device. Stx2a was serially diluted in buffer from 500 ng/mL to 0.1 ng/mL, and three independent test strips at each dilution were evaluated. The performance of the LFA was validated by resolution of the control line with a total test time of 5 min. Qualitative detection of Stx2a by visual inspection revealed a dose-dependent increase in test line density with an LOD of 0.1 ng/mL (Figure 2A). To further evaluate the relationship between Stx2a concentration and LFA performance, we performed densitometric analysis of the test line at each dilution in triplicate. The density plot resulted in a sigmoidal-shaped curve that revealed a plateau in test line density at concentrations >100 ng/mL (Figure 2B). Regression analysis for Stx2a test line densities <100 ng/mL showed an optimal linear relationship (*R^2^* = 0.99) of this LFA at Stx2a concentrations between 25 ng/mL and 2.5 ng/mL (Figure 2B; solid line). These results suggest that this LFA could be used to make a quantitative determination of Stx2a concentration from a test line density read from a Stx2a standard curve established between 20 ng/mL and 2.5 ng/mL.

**Figure 3:**
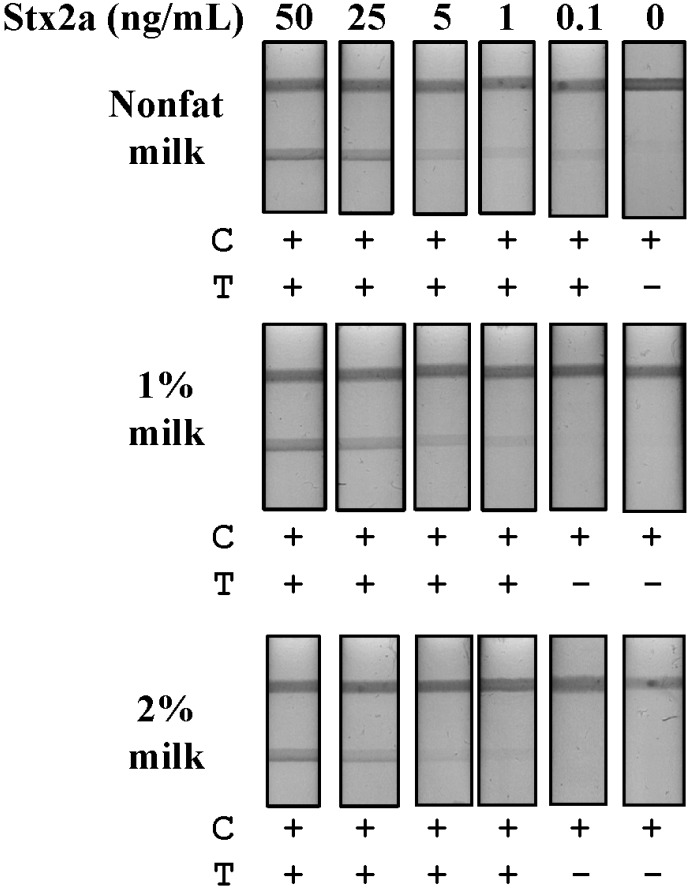
Detection of Stx2a from spiked milk matrices by LFA. Samples of nonfat, 1% and 2% milk were spiked with purified Stx2a (50–0.1 ng/mL) and centrifuged to remove lipids. The defatted samples were then diluted 10-fold in PBS and evaluated for Stx2a detection by LFA. T, test line; C, control line; +, positive test result; −, negative test result.

Detection of Stx2a in spiked food matrices: Milk, lettuce and ground beef all represent food matrices implicated in STEC outbreaks [5,27]. First, we evaluated commercially obtained milk containing three concentrations of fat (nonfat, 1% and 2%) without an Stx2a spike by LFA and observed both slow lateral flow and non-specific binding to our test line, even after centrifugation or with the use of additional detergents or blocking agents (data not shown). However, simple dilution of the raw milk product (1:10 in PBS) was sufficient to eliminate non-specific binding to the test line and to facilitate fluid flow. As a diluted sample, fluid flow was inversely related to the fat content of the milk, with the fastest flow occurring with nonfat milk and the slowest with 2% milk samples. Next, we spiked milk samples with Stx2a at five concentrations (50–0.1 ng/mL), then diluted them in PBS and evaluated Stx2a detection by LFA (Figure 3). Nonfat milk as a substrate resulted in the greatest Stx2a detection sensitivity with an LOD of 0.1 ng/mL (Figure 3A). The 1% and 2% milk substrates showed equivalent Stx2a detection sensitivities with LODs of 1 ng/mL (Figure 3B). In the 2% milk samples, aggregation of gold conjugate, evidenced by a reddish line at the base of the device following the complete resolution of the test, also accompanied reduced fluid flow (data not shown). This resulted in a somewhat weaker overall visual signal in the 2% milk samples, as compared to those at equal Stx2a concentrations in 1% milk, but sufficient for qualitative determination. These results demonstrate that a simple dilution of milk samples is sufficient to eliminate non-specific test line binding, to improve sample fluid flow and results in sensitive Stx2a detection using an LFA.

**Figure 4:**
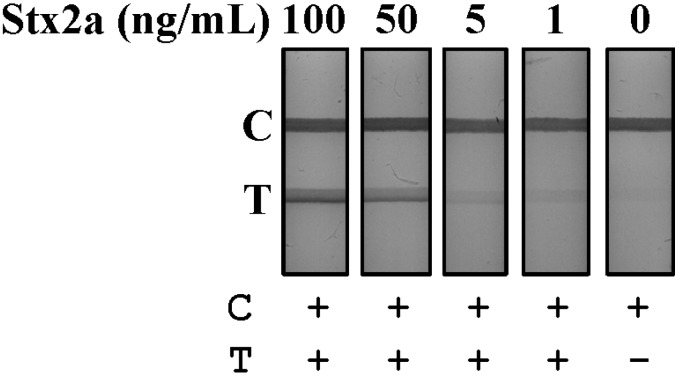
Detection of Stx2a from spiked lettuce samples by LFA. Lettuce samples were chopped, suspended in PBS and spiked with purified Stx2a (100–1 ng/mL). Samples were then mixed, centrifuged and the resulting supernatants evaluated for Stx2a detection using the LFA. T, test line; C, control line; +, positive test result; −, negative test result.

Leafy greens can become contaminated with STEC during growth or harvest, as fields are typically fertilized with manure or inadvertently exposed to water runoff contaminated with feces, both potential reservoirs of STEC bacteria [28,29]. Samples of lettuce (0.1 g) were chopped, placed in 1 mL of PBS and spiked with four concentrations of purified Stx2a (100–1 ng/mL). Spiked samples were vortexed, then centrifuged for 15 min at 12,000× *g* at 4 °C to remove solids and the supernatant tested by LFA (Figure 4). The lateral flow rate of lettuce supernatants was equivalent to buffer with the LFA resolving in 1 min at an LOD of 1 ng/mL.

**Figure 5:**
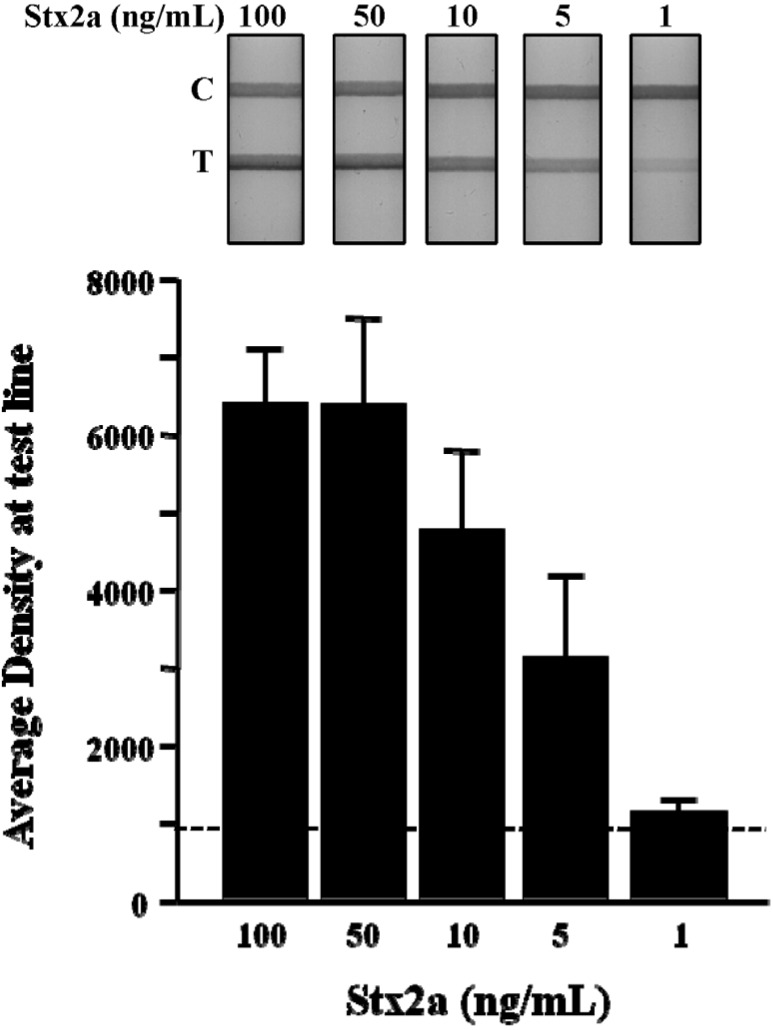
Detection of Stx2a from spiked ground beef by LFA. Ground beef was suspended in PBS and spiked with purified Stx2a (100 to 1 ng/mL). The samples were mixed, centrifuged to reduce fat content and the resulting supernatant evaluated for Stx2a detection by LFA (**top panel**). Density measurements were obtained at the test line from three independent experiments and data plotted as the mean (±SEM) at the test line for each Stx2a dilution (**bottom panel**). The dotted line represents the average density at the test line for beef without an Stx2a spike plus three standard deviations. T, test line; C, control line.

Ground beef is a common source of STEC contamination and is most frequently implicated in serious disease outbreaks [5,6]. We prepared samples of 90% lean ground beef (0.1 g) in 1 mL of PBS, then spiked samples with four concentrations of purified Stx2a (100–1 ng/mL). This material was vortexed, and the suspension was centrifuged for 15 min at 12,000× *g*, 4 °C. The supernatant was decanted and tested directly for Stx2a detection without dilution using the LFA (Figure 5A). Fluid flow from spiked beef samples was somewhat slower compared to milk and lettuce samples, most likely due to the high protein and lipid content of the sample, and test line resolution was complete in <10 min. Three independent experiments were performed at each spike concentration of Stx2a, and the mean ± SEM test line densities were plotted (Figure 5B). The dashed line represents the average density of the test line for beef samples without an Stx2a spike, plus three standard deviations. These data show the dose-density relationship of Stx2a-spiked beef samples at the test line of the LFA and demonstrate that Stx2a spikes of ≥5 ng/mL were sufficient for reliable detection of the toxin contaminant.

## 4. Discussion

STEC contamination of food is a primary source of infection, and Stxs play a central role in disease pathogenesis that can result in serious illness and death [8]. Sensitive STEC detection methods are essential to identifying a source of contamination and limiting its spread during food production and preparation. Toward this aim, we have developed a sensitive and rapid LFA that can detect Stx1 and six Stx2 variants produced by STEC bacteria. Our device uses two new mAbs for the capture and detection of Stx and a colorimetric gold-conjugate for visual resolution of a test line for positive confirmation. We demonstrate the utility of this LFA for the detection of Stxs from bacterial culture supernatants and food matrices with LODs of ~1 ng/mL. Our LFA provides a rapid and simple-to-use Stx detection technology for the evaluation of bacterial cultures or suspect samples contaminated with STEC.

Current food safety measures for *E. coli* O157:H7 detection utilize a “hold and test” procedure, where product is held at the production facility ~2 days until test results are complete. These tests are based on the inability of *E. coli* O157:H7 to ferment sorbitol in selective growth media [9]. More recently, pathogenic non-O157 STEC strains, which cannot be identified by this method, have been isolated, necessitating the development of alternative means of detection to ensure food safety [10]. These include: pulsed-field gel electrophoresis (PFGE) for strain identification, polymerase chain reaction (PCR) to genetically determine the expression of virulence genes, the Vero cell assay to determine cytotoxicity and enzyme immunoassays (EIA) using selective antibodies to identify Stx and other gene products [30,31]. The application of this simple LFA following *E. coli* enrichment from target samples could be used to rapidly identify the presence of Stx production irrespective of the bacterial strain.

The performance of our Stx LFA is contingent upon the use of a pair of previously described high affinity mAbs (Stx2-1 and Stx2-2) in a sandwich ELISA format [20]. These mAbs do not discriminate between Stx1 and six of the Stx2 variants, recognizing them equally at the test line of the LFA. Our Stx LFA demonstrates high sensitivity to purified toxin and broad specificity across Stx variants produced by STEC serotypes. The broad detection capability of our LFA for Stxs is of great utility, as it is increasingly clear that pathogenic strains of STEC can produce any of the Stx protein variants [32]. Importantly, the direct detection of Stx by LFA serves as a rapid confirmation of an STEC contamination.
